# Supplementary material for: Heat adaptation of phage T7 under an extended genetic code
Source: Virus Evol. 2021 Dec 1;7(2):veab100. doi: 10.1093/ve/veab100 (PMC8923235; doi:10.1093/ve/veab100)
Supplement: veab100_Supp [file veab100_supp.zip › Sequencing_Analysis.zip!/Sequencing_Analysis/S2/evidence/MC_PLOT_38.html]

BRESEQ :: Evidence


| Predicted mutation | | | | | | |
| --- | --- | --- | --- | --- | --- | --- |
| evidence | seq id | position | mutation | annotation | gene | description |
| MC JC | NC\_001604.1 | 1,253 | Δ1,489 bp | intergenic (–/–) | – / – | –/– |

| Missing coverage evidence... | | | | | | | | | | |
| --- | --- | --- | --- | --- | --- | --- | --- | --- | --- | --- |
|  |  |  | seq id | start | end | size | ←reads | reads→ | gene | description |
| \* | \* | ÷ | NC\_001604.1 | 1253 | 2741 | 1489 | 5746 [2] | [2] 5753 | *–/–* | –/– |

| New junction evidence | | | | | | | | | | | |
| --- | --- | --- | --- | --- | --- | --- | --- | --- | --- | --- | --- |
|  | | seq id | position | reads (cov) | reads (cov) | score | skew | freq | annotation | gene | product |
| \* | ? | NC\_001604.1 | = 1252 | 2 (0.000) | 5642 (1.100) | 443/580 | 0.0 | 100.0% | intergenic (–/–) | *–/–* | –/– |
|  | ? | NC\_001604.1 | 2742 = | 0 (0.000) |  |  |  |  | intergenic (–/–) | *–/–* | –/– |
